# Ultrasound-Based Radiomics Can Classify the Etiology of Cervical Lymphadenopathy: A Multi-Center Retrospective Study

**DOI:** 10.3389/fonc.2022.856605

**Published:** 2022-05-17

**Authors:** Yajing Liu, Jifan Chen, Chao Zhang, Qunying Li, Hang Zhou, Yiqing Zeng, Ying Zhang, Jia Li, Wen Xv, Wencun Li, Jianing Zhu, Yanan Zhao, Qin Chen, Yi Huang, Hongming Li, Ying Huang, Gaoyi Yang, Pintong Huang

**Affiliations:** ^1^ Department of Ultrasound in Medicine, The Second Affiliated Hospital of Zhejiang University School of Medicine, Zhejiang University, Hangzhou, China; ^2^ Department of Ultrasound, Hangzhou Red Cross Hospital, Hangzhou, China; ^3^ Department of Ultrasound, Sichuan Academy of Medical Sciences and Sichuan Provincial People’s Hospital, School of Medicine, Chengdu, China; ^4^ Department of Ultrasound Diagnosis, Xi’an Chest Hospital, Xi’an, China; ^5^ Physical Diagnosis Department, Infectious Disease Hospital of Heilongjiang Province, Harbin, China; ^6^ Department of Ultrasound, Shengjing Hospital of China Medical University, Liaoning Province, Shenyang, China; ^7^ Research Center of Ultrasound in Medicine and Biomedical Engineering, The Second Affiliated Hospital of Zhejiang University School of Medicine, Zhejiang University, Hangzhou, China

**Keywords:** radiomics, cervical lymph nodes tuberculosis, cervical lymphoma, reactive lymph node hyperplasia, metastatic lymph node

## Abstract

Medical diagnostic imaging is essential for the differential diagnosis of cervical lymphadenopathy. Here we develop an ultrasound radiomics method for accurately differentiating cervical lymph node tuberculosis (LNTB), cervical lymphoma, reactive lymph node hyperplasia, and metastatic lymph nodes especially in the multi-operator, cross-machine, multicenter context. The inter-observer and intra-observer consistency of radiomics parameters from the region of interest were 0.8245 and 0.9228, respectively. The radiomics model showed good and repeatable diagnostic performance for multiple classification diagnosis of cervical lymphadenopathy, especially in LNTB (area under the curve, AUC: 0.673, 0.662, and 0.626) and cervical lymphoma (AUC: 0.623, 0.644, and 0.602) in the whole set, training set, and test set, respectively. However, the diagnostic performance of lymphadenopathy among skilled radiologists was varied (Kappa coefficient: 0.108, *p < 0.001). The diagnostic performance of radiomics is comparable and more reproducible compared with those of skilled radiologists. Our study offers a more comprehensive method for differentiating LNTB, cervical lymphoma, reactive lymph node hyperplasia, and metastatic LN.

## Introduction

Lymph nodes (LN), which are distributed throughout the body, are an important immune organ (1). There are many reasons for the swelling of the shallow lymph nodes, including local or systemic inflammatory infections, tumors, blood system diseases, *etc.* (2). An accurate diagnosis of cervical lymphadenopathy plays an important role in clinical management. Currently, the diagnostic assessment of cervical lymphadenopathy focuses on distinguishing between benign and malignant LNs. However, there are no highly accurate methods for the differential diagnosis of LN tuberculosis (LNTB), cervical lymphoma, metastatic LN, and reactive LN hyperplasia (3–5).

Globally, tuberculosis (TB) is one of the 10 leading causes of death (6, 7). About one in four people worldwide infected with *Mycobacterium tuberculosis* are susceptible to developing TB disease (8). Cervical LNTB is one of the most common types of extrapulmonary TB, the representative symptom of which is lymph node enlargement (9). Its treatment mainly depends on medical interventions. Ultrasound has irreplaceable value for the pretreatment diagnosis and treatment efficacy assessment of LNTB (10–12). Cervical lymphoma is a malignant tumor characterized by enlarged LN. Cervical metastatic LN refers to regional nodal involvement by cancer in the head and neck and other organs in the body. Identifying suspicious lymph node metastases *via* preoperative ultrasound is critical to clinical management. Reactive LN hyperplasia, with the typical symptom of enlargement of lymph tissue, is benign and is usually stimulated by different kinds of antigens. There are some overlapping clinical and imaging appearances for the differential diagnosis of cervical lymphadenopathy.

Recently, owing to the rapid development of ultrasound technology and equipment, ultrasound has become the first-line method for diagnosing superficial lymph node lesions. Ultrasound is a non-invasive method with high penetration depth and has the ability to conduct real-time imaging (13–16). It can also provide multiparameter information, such as lymph node structure, elasticity, and blood perfusion, which is beneficial for the accurate assessment of cervical lymphadenopathy (17). The morphological and structural features of LN are usually derived from ultrasound images by experienced radiologists. However, the diagnostic accuracy of visual observation is subjective and is largely dependent on the radiologist’s experience, which limits the accuracy and repeatability of diagnosis.

Radiomics, as an excellent computer-aided diagnosis method, especially in combination with multicenter clinical information, has great potential for disease identification, diagnosis, and prognosis evaluation (18–20). Radiomics is a process through which imaging features are extracted from medical images in a high-throughput way, transformed into high-resolution, mineable data, and quantitatively analyzed (21). Radiomics has shown good ability for the detection of lymphoma and metastatic LN in a binary classification manner (22–24). However, few studies explored whether radiomics has good capability in the multi-classification discrimination of LNTB, cervical lymphoma, reactive LN hyperplasia, and metastatic LN (25, 26).

Herein a multicenter, retrospective, automated radiomics model based on superficial lymph node B-mode images was built to achieve accurate diagnosis for four typical causes of cervical lymphadenopathy in this study.

## Materials and Methods

### Design

This retrospective, multicenter study was approved by the institutional ethics committees of all medical centers involved. The ethics committee waived the requirement of written informed consent for participation. The identifier NCT04497714 was assigned to our study at ClinicalTrials.gov. In this study, 1,105 patients with clinicopathologically confirmed LNTB, cervical lymphoma, reactive LN hyperplasia, or metastatic LN were collected from six hospitals from January 2017 to January 2020. These hospitals were The Second Affiliated Hospital of Zhejiang University School of Medicine, Hangzhou Red Cross Hospital, Xi’an Chest Hospital, Sheng Jing Hospital of China Medical University, Sichuan Provincial People’s Hospital, and Infectious Disease Hospital of Heilongjiang Province. We reviewed the patients’ electronic medical records, and all patients were enrolled with pathological reports.

### Patients

The cervical lymph node ultrasound images were collected from six hospitals with jpeg, jpg, or bmp format. These images had been generated by different models of ultrasound instrument (Philips, Mindray, and GE Healthcare), so image quality control was performed before the data processing procedures. Ultrasound images were taken before the puncture. The time interval between the pathological results obtained after the ultrasound images were taken was 24 h. All enrolled patients were pathologically confirmed with LNTB, cervical lymphoma, reactive lymph nodes hyperplasia, and metastatic LN.

The inclusion criteria were as follows (1): cervical lymphadenopathy was confirmed by pathological examination and (2) included at least two ultrasound static images of the target lymph node (transverse and longitudinal images) before the puncture.

The exclusion criteria were as follows (1): patients with HIV (2), the lesion was not confirmed by pathological examination, and (3) the quality of the ultrasound images was poor.

The general clinical data of patients (age, gender, lesion size, and pathological imaging results of the lesions) were recorded.

### Procedures

The patient was in the supine or lateral position to expose the neck. The neck ultrasound examination was performed by experienced radiologists. First, gray-scale ultrasound imaging was performed to observe the size, boundary, hilum of lymph node, and the longitudinal diameter (L) and short diameter (S) of each lymph node on the maximum longitudinal section. After collecting the image data, three skilled radiologists with at least 5 years of experience in superficial LN ultrasound examination interpreted the randomly ordered cervical lymph node ultrasound images blindly. The diagnostic performance of each radiologist in cervical lymphadenopathy was compared with the pathological results.

In the ultrasound-based radiomics process, the collected data were randomly assigned to a training set (60%) and a test set (40%). The lymph node boundaries were delineated by two radiologists who were unaware of the purpose of the study. The inter- and intra-observer consistency values of the radiomics parameters extracted from the region of interest (ROI) were tested by calculating the intraclass correlation coefficient (ICC). The radiomics parameters were extracted using a MATLAB (R2020a) package of radiomics analysis from Github (github.com/mvallieres/radiomics), and the statistical modeling was performed by R software (version 3.6.2). The quantitative features of radiomics were extracted from B-mode ultrasound images, including 11 statistical features, 26 gray-level co-occurrence matrix (GLCM) features, 13 gray-level run-length matrix (GLRLM) features, 16 gray-level size zone matrix (GLSZM) features, 16 gray-level distance zone matrix (GLDZM) features, 5 neighborhood gray-tone difference matrix (NGTDM) features, and 17 neighboring gray-level dependence matrix (NGDLM) features. The least absolute shrinkage and selection operator (LASSO) algorithm was used to select the features, and the support vector machine (SVM) algorithm was applied to differentiate four causes of cervical lymphadenopathy. In this study, a multiple classification of LNTB, cervical lymphoma, reactive LN hyperplasia, and metastatic LN was performed using LASSO–Vote–SVM model, a customized version of the LASSO–SVM model, to obtain the category that the sample belongs to. The receiver operating characteristic (ROC) curves of the radiomics model and of the three skilled radiologists were plotted, and the Kappa coefficient within radiologists was calculated. The area under the curve (AUC), sensitivity, and specificity of the radiomics model and those of the radiologists were calculated based on R packages “pROC” and “ROCit”. McNemar’s test was used for comparing the sensitivity and specificity between the radiomics model and the radiologists. Delong’s test was used for comparing the AUCs among radiologists and the radiomics model. Statistical significance was defined as *p*-value <0.05, and the multi-comparison *p*-value was adjusted by Bonferroni correction.

## Results

### Patients’ Distribution and Demographic Characteristics

**Figure 1:**
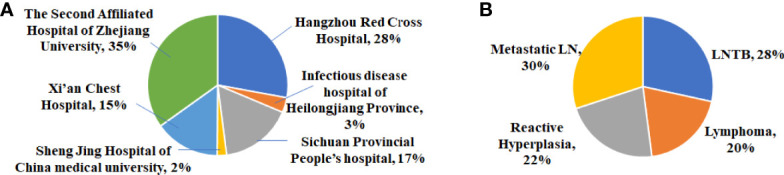
**(A)** Distribution of cervical lymphadenopathy in six medical centers. **(B)** Distribution of four categories of cervical lymphadenopathy.

**Figure 2:**
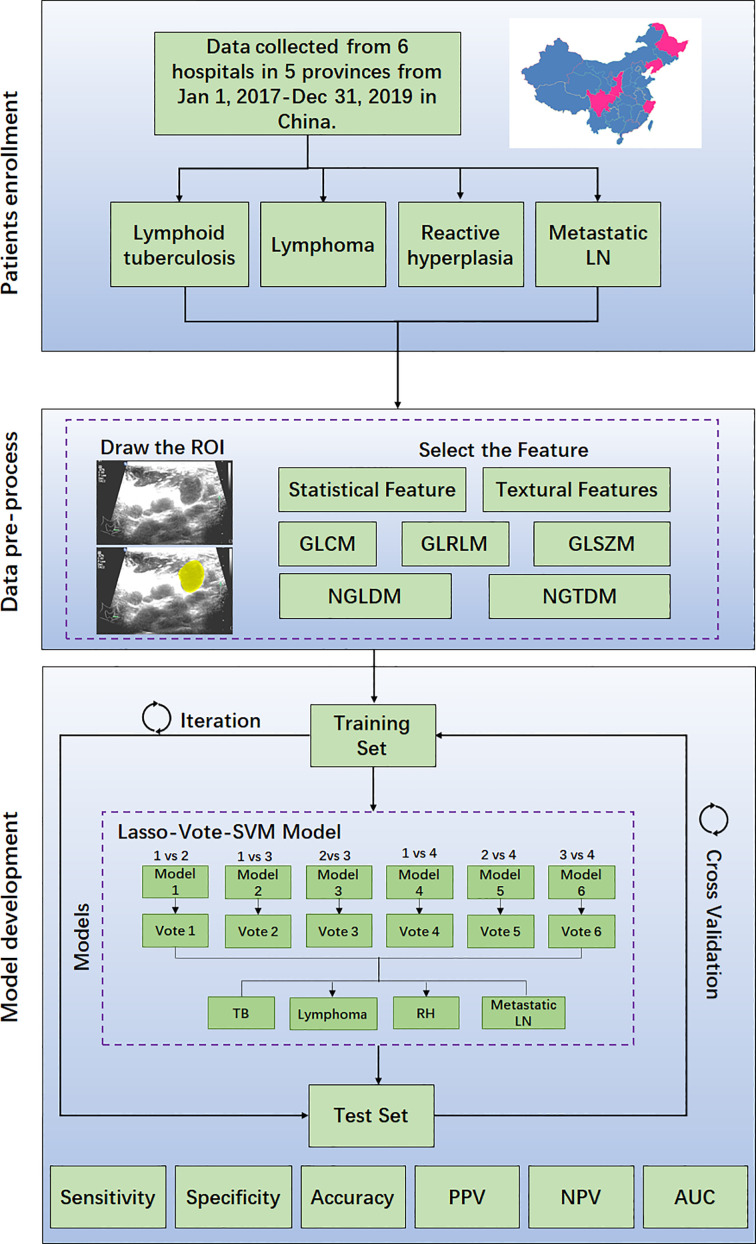
Enrollment flow chart of this study.

Between January 1, 2017 and January 1, 2020, 1,245 cervical lymphadenopathy ultrasound images were obtained from six different hospitals (**Figure 1**). The average age of the enrolled patients was 48.1 ± 18.9, and 579 male patients and 526 female patients were enrolled (**Table 1**). **Figure 2** depicts the workflow of patient enrollment, data pre-processing, and radiomics model development for multiple classification diagnosis of cervical lymphadenopathy. After the image quality control step, 1,105 images (one image per patient) were retained for our study, including 314 images (28% of total) with LNTB, 216 images (20% of total) with cervical lymphoma, 332 images (30% of total) with metastatic LN, and 243 images (22% of total) with reactive LN hyperplasia (**Table 1**). Among the 1,105 total images, 308 were obtained from Hangzhou Red Cross Hospital, 38 were obtained from Infectious Disease Hospital of Heilongjiang Province, 184 were obtained from Sichuan Provincial People’s Hospital, 24 were obtained from Sheng Jing Hospital of China Medical University, 166 images were obtained from Xi’an Chest Hospital, and 385 were obtained from The Second Affiliated Hospital of Zhejiang University School of Medicine.

**Table 1:** Case distribution in six medical centers.

Variables	Hangzhou Red Cross Hospital	Heilongjiang Infectious Disease Control Hospital	Sichuan Provincial Cancer Hospital	Sheng Jing Hospital	Xi’an Chest Hospital	The Second Affiliated Hospital of Zhejiang University		All Center
Number of patients	308	38	184	24	166	385		1,105
Lymph node tuberculosis	144	10	52	2	66	40		314
Lymphoma	58	1	60	5	2	90		216
Reactive hyperplasia	54	1	38	10	82	58		243
Metastatic lymph node	52	26	34	7	16	197		332
Male	143	19	85	9	90	233		579
Female	165	19	99	15	76	152		526
Age	44.4 ± 20.0	48.6 ± 17.2	46.7 ± 19.9	42.7 ± 15.2	38.4 ± 17.2	56.1 ± 15.3	48.1 ± 18.9	

### Inter-/Intra-Observer Consistency of the Radiomics Parameters From Delineated ROI in Lymphadenopathy

**Figure 3:**
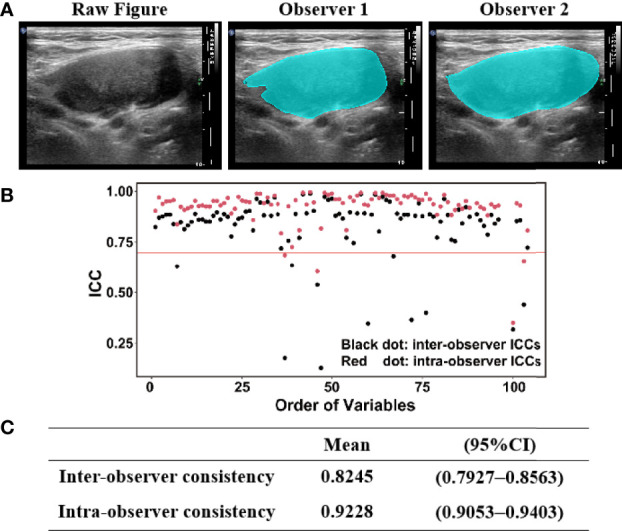
**(A)** ROI (region of interest) was delineated according to the lymph node boundary by two radiologists. **(B,C)** Intraclass correlation coefficient (ICC) scores for inter- observer and intra-observer measurements

The ROI was delineated blindly according to the lymph node boundary by two colleagues (CZ and YZ) who were unaware of the purpose of the study (**Figure 3A**). Using MATLAB software, 104 features were extracted, including 11 statistical features, 26 GLCM features, 13 GLRLM features, 16 GLSZM features, 16 GLDZM features, 5 NGTDM features, and 17 NGDLM features. The ICCs were used to evaluate the inter-/intra-observer repeatability of the radiomics parameters from the delineated ROI (**Figure 3B**). The inter-observer and intra-observer consistency were 0.8245 (0.7927–0.8563) and 0.9228 (0.9053–0.9403), respectively (**Figure 3C**). Fifteen features with ICC lower than 0.7 were deleted in the model building step to obtain a repeatability result (**Figure 3B**).

### Diagnostic Performance of Lymphadenopathy in the Radiomics Model and the Radiologists

The whole dataset was randomly assigned into a training set consisting of 663 (60%) cases and a testing set consisting of 442 (40%) cases. For our study, the multinomial LASSO–logistic regression model and the LASSO–Vote–SVM model were selected. In the training set, the two machine learning models had a similar diagnostic performance, but in the test set, the accuracy of the LASSO–Vote–SVM model was better than the LASSO–logistic regression model (0.666, 0.655–0.677; 0.783, 0.773–0.793; 0.773, 0.764–0.784; and 0.685, 0.676–0.693 for LNTB, cervical lymphoma, reactive LN hyperplasia, and metastatic LN, respectively) (**Supplementary Table S1**).

Based on these results, we chose the LASSO–Vote–SVM model to calculate the radiomics model’s performance for differentially diagnosing cervical lymphadenopathy. Besides this, three skilled radiologists read the whole image set blindly, and the results were collected. **Tables 2**, **3** show the sensitivity, specificity, accuracy, negative predictive value, and positive predictive value of the radiomics model and the radiologists (R1, R2, and R3) in the diagnosis of LNTB, cervical lymphoma, reactive LN hyperplasia, and metastatic LN.

**Table 2:** Comparison between radiomics model and senior radiologists in training set.

Disease	Index	Radiomics model		R1 (95%CI)		R2 (95%CI)		R3 (95%CI)	
		Median	95%CI	Median	95%CI	Median	95%CI	Median	95%CI
Lymph node tuberculosis	Sensitivity	0.599	(0.517–0.680)	0.301	(0.259–0.337)	0.375	(0.315–0.403)	0.025	(0.010–0.041)
	Specificity	0.763	(0.710–0.806)	0.898	(0.886–0.912)	0.852	(0.834–0.875)	0.980	(0.972–0.987)
	Accuracy	0.716	(0.670–0.754)	0.727	(0.711–0.747)	0.718	(0.693–0.740)	0.707	(0.691–0.725)
	Negative predictive value (NPV)	0.824	(0.805–0.852)	0.763	(0.744–0.781)	0.771	(0.755–0.792)	0.716	(0.700–0.733)
	Positive predictive value (PPV)	0.502	(0.440–0.566)	0.541	(0.486–0.599)	0.5	(0.445–0.552)	0.325	(0.156–0.543)
Lymphoma	Sensitivity	0.328	(0.210–0.482)	0.338	(0.285-0.391)	0.351	(0.299-0.398)	0.093	(0.062-0.111)
	Specificity	0.931	(0.895-0.968)	0.930	(0.917-0.940)	0.834	(0.804-0.853)	0.985	(0.978-0.991)
	Accuracy	0.817	(0.790-0.849)	0.816	(0.798-0.834)	0.738	(0.713-0.754)	0.812	(0.791-0.825)
	NPV	0.854	(0.835-0.881)	0.855	(0.835-0.868)	0.842	(0.823-0.856)	0.819	(0.800-0.833)
	PPV	0.549	(0.436-0.651)	0.534	(0.473-0.607)	0.331	(0.282-0.399)	0.6	(0.469-0.706)
Reactive Hyperplasia	Sensitivity	0.441	(0.341-0.537)	0.467	(0.418-0.506)	0.256	(0.212-0.290)	0.593	(0.554-0.649)
	Specificity	0.903	(0.871-0.928)	0.782	(0.767-0.805)	0.918	(0.906-0.937)	0.624	(0.601-0.650)
	Accuracy	0.798	(0.769-0.825)	0.713	(0.693-0.734)	0.772	(0.755-0.793)	0.620	(0.598-0.637)
	NPV	0.851	(0.832-0.870)	0.839	(0.821-0.858)	0.815	(0.798-0.833)	0.847	(0.827–0.873)
	PPV	0.558	(0.491–0.618)	0.379	(0.343–0.407)	0.465	(0.412–0.532)	0.312	(0.280–0.338)
Metastatic LN	Sensitivity	0.676	(0.594–0.738)	0.693	(0.665–0.743)	0.683	(0.655–0.729)	0.678	(0.632–0.715)
	Specificity	0.770	(0.696–0.823)	0.655	(0.635–0.682)	0.625	(0.602–0.645)	0.545	(0.514–0.566)
	Accuracy	0.742	(0.705–0.789)	0.667	(0.656–0.685)	0.641	(0.622–0.667)	0.583	(0.562–0.603)
	NPV	0.847	(0.819–0.877)	0.834	(0.814–0.861)	0.821	(0.800–0.849)	0.795	(0.777–0.818)
	PPV	0.553	(0.519–0.625)	0.466	(0.446–0.488)	0.439	(0.411–0.465)	0.390	(0.360–0.418)

**Table 3:** Comparison between radiomics model and senior radiologists in test set.

Disease	Index	Radiomics model		R1 (95%CI)		R2 (95%CI)		R3 (95%CI)	
		Median	95%CI	Median	95%CI	Median	95%CI	Median	95%CI
Lymph node tuberculosis	Sensitivity	0.496	(0.317–0.646)	0.297	(0.247–0.353)	0.362	(0.321–0.440)	0.026	(0–0.049)
	Specificity	0.736	(0.634–0.830)	0.891	(0.869–0.909)	0.852	(0.817–0.879)	0.979	(0.968–0.991)
	Accuracy	0.667	(0.619–0.703)	0.724	(0.693–0.748)	0.71	(0.678–0.748)	0.71	(0.683–0.735)
	Negative predictive value (NPV)	0.786	(0.752–0.824)	0.764	(0.736–0.792)	0.775	(0.745–0.801)	0.719	(0.692–0.742)
	Positive predictive value (PPV)	0.426	(0.362–0.492)	0.517	(0.432–0.590)	0.495	(0.422–0.580)	0.348	(0–0.530)
Lymphoma	Sensitivity	0.225	(0.074–0.410)	0.313	(0.239–0.399)	0.342	(0.265–0.413)	0.08	(0.050–0.125)
	Specificity	0.918	(0.853–0.976)	0.927	(0.912–0.948)	0.827	(0.799–0.873)	0.986	(0.977–0.997)
	Accuracy	0.783	(0.754–0.826)	0.805	(0.779–0.833)	0.735	(0.711–0.772)	0.807	(0.787–0.838)
	NPV	0.833	(0.804–0.862)	0.844	(0.825–0.874)	0.836	(0.815–0.864)	0.813	(0.792–0.840)
	PPV	0.426	(0.318–0.563)	0.522	(0.408–0.625)	0.338	(0.241–0.404)	0.586	(0.412–0.784)
Reactive hyperplasia	Sensitivity	0.387	(0.283–0.552)	0.451	(0.388–0.527)	0.254	(0.205–0.321)	0.611	(0.538–0.665)
	Specificity	0.879	(0.802–0.933)	0.784	(0.750–0.808)	0.923	(0.895–0.940)	0.624	(0.586–0.658)
	Accuracy	0.771	(0.738–0.803)	0.71	(0.679–0.742)	0.776	(0.745–0.802)	0.618	(0.592–0.651)
	NPV	0.834	(0.815–0.869)	0.836	(0.807–0.862)	0.813	(0.786–0.839)	0.848	(0.811–0.877)
	PPV	0.474	(0.366–0.603)	0.368	(0.321–0.423)	0.486	(0.391–0.566)	0.308	(0.269–0.355)
Metastatic lymph node	Sensitivity	0.606	(0.486–0.678)	0.693	(0.620–0.735)	0.677	(0.611–0.721)	0.669	(0.611–0.736)
	Specificity	0.729	(0.627–0.813)	0.66	(0.620–0.691)	0.625	(0.596–0.659)	0.535	(0.504–0.580)
	Accuracy	0.692	(0.632–0.730)	0.67	(0.643–0.685)	0.643	(0.604–0.671)	0.577	(0.548–0.610)
	NPV	0.81	(0.769–0.842)	0.831	(0.792–0.860)	0.818	(0.779–0.849)	0.795	(0.758–0.820)
	PPV	0.493	(0.432–0.550)	0.462	(0.430–0.493)	0.436	(0.398–0.480)	0.382	(0.341–0.425)

For LNTB, the AUC of radiomics model is 0.673 (0.637–0.710), 0.662 (0.613–0.710), and 0.626 (0.567–0.684) in the whole set, training set, and test set, respectively. For lymphoma, the AUC of the radiomics model is 0.623 (0.579–0.666), 0.644 (0.613–0.699), and 0.602 (0.533–0.674) in the whole set, training set, and test set, respectively. For reactive hyperplasia, the AUC of the radiomics model is 0.655 (0.614–0.695), 0.661 (0.609–0.714), and 0.602 (0.536–0.668) in the whole set, training set, and test set, respectively. For metastatic LN, the AUC of the radiomics model is 0.708 (0.673–0.743), 0.717 (0.672–0.761), and 0.683 (0.626–0.740) in the whole set, training, and test set, respectively (**Table 4** and **Supplementary Table S2**).

**Table 4:** Area under the curve between the radiomics model and the senior radiologists in the test set.

Data set	Disease	Radiomics	R1	R2	R3
Test set	Lymph node tuberculosis	0.626 (0.567–0.684)	0.582 (0.522–0.640)	0.605 (0.546–0.664)	0.502 (0.443–0.560)
	Lymphoma	0.602 (0.533–0.672)	0.638 (0.569–0.707)	0.594 (0.525–0.664)	0.541 (0.472–0.610)
	Reactive hyperplasia	0.602 (0.536–0.668)	0.646 (0.581–0.711)	0.580 (0.514–0.646)	0.617 (0.551–0.682)
	Metastatic lymph node	0.683 (0.626–0.740)	0.700 (0.644–0.757)	0.652 (0.593–0.710)	0.625 (0.566–0.684)

In **Supplementary Table S3**, 177 (16.0%), 134 (12.1%), 299 (27.1%), and 499 (44.8%) lesions were diagnosed as LNTB, cervical lymphoma, reactive LN hyperplasia, and metastatic LN, respectively, by radiologist 1 (R1). In total, 233 (21.1%), 225 (20.4%), 130 (11.8%), and 516 (46.7%) lesions were diagnosed as LNTB, lymphoma, reactive hyperplasia, and metastatic LN, respectively, by radiologist 2 (R2). Moreover, 24 (2.2%), 32 (2.9%), 469 (42.5%), and 578 (52.4%) lesions were diagnosed as LNTB, cervical lymphoma, reactive LN hyperplasia, and metastatic LN, respectively, by radiologist 3 (R3). A low agreement among the three radiologists in the diagnosis of lymphadenopathy was found in this study, especially in LNTB and lymphoma. The Kappa coefficient was 0.108 (*p < 0.001). For LNTB, the AUC of the three radiologists varies from 0.502 (0.443–0.560) to 0.615 (0.565–0.664) in the whole set, training set, and test set. For lymphoma, the AUC of the three radiologists varies from 0.534 (0.478–0.589) to 0.638 (0.569–0.707) in the whole set, training set, and test set (**Table 4** and **Supplementary Table S2**).

### Comparison Among the Radiomics Model and the Radiologists in the Multiple Classification Diagnosis of Cervical Lymphadenopathy

**Figure 4:**
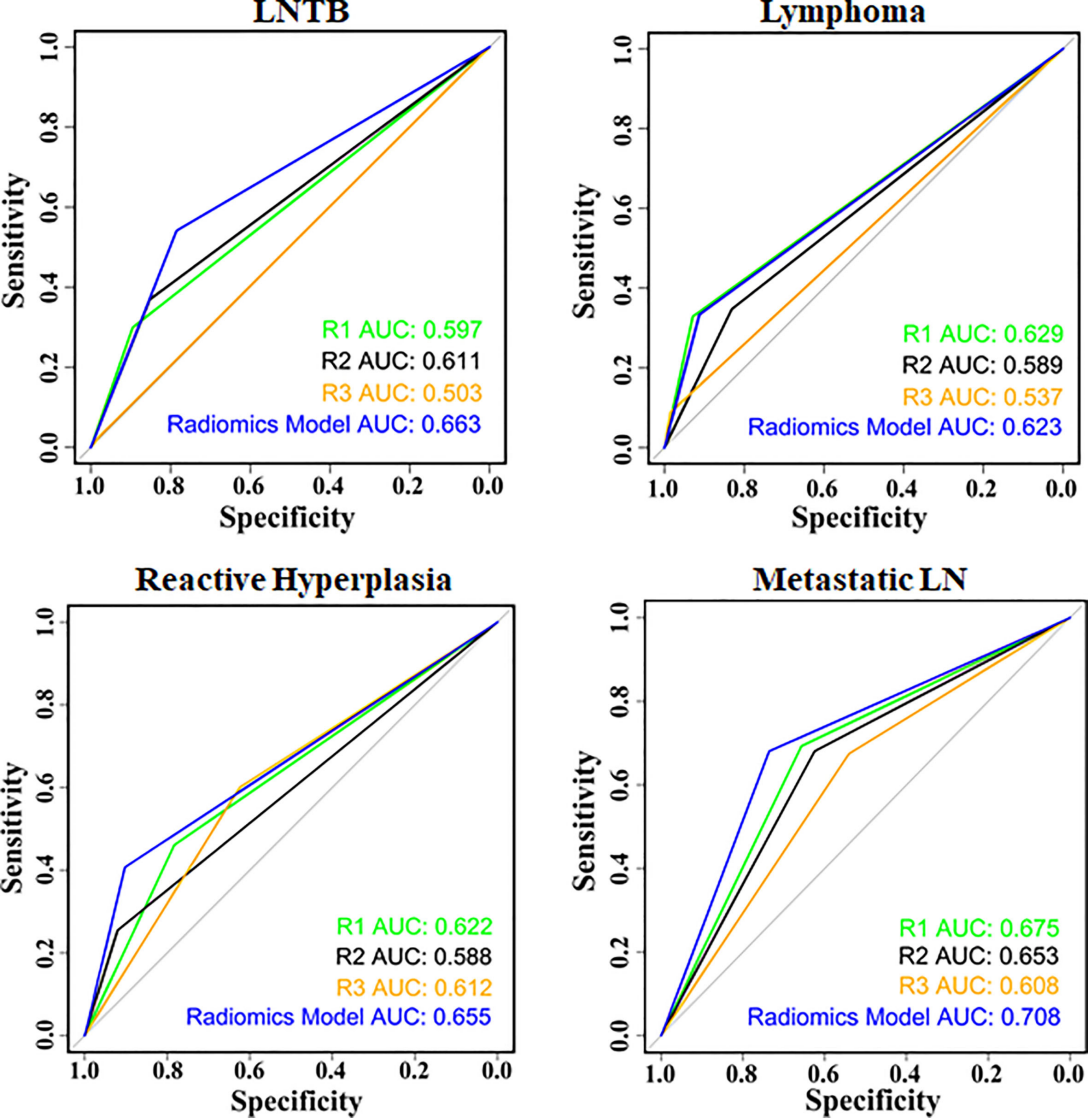
Receiver operating characteristic between the radiomics model and radiologists in the whole set.

Then, we compared the radiomics model (LASSO–Vote–SVM) with the three skilled radiologists (with more than 5 years of experience in the diagnosis of cervical lymphadenopathy by ultrasound) for the multiple classification diagnosis of cervical lymphadenopathy. The ROC plots of the radiomics model and the skilled radiologists are shown in **Figure 4** and **Supplementary Figures S1**, **S2**. The AUC of the radiomics model in the diagnosis of LNTB was statistically higher than the AUCs of radiologists 1–3 in the whole set, and it was statistically higher than radiologist 3 in the training set and the test set (all *p* < 0.008) (**Supplementary Table S4**). The radiomics model showed higher sensitivity and specificity compared with radiologists 1–3 in the whole set, training set, and test set (**Supplementary Tables S5, S6**) in the diagnosis of LNTB. This performance for LNTB shows that radiomics might have a potent differentiating capability in LNTB and could assist radiologists to make more accurate diagnoses.

For lymphoma, the AUC of the radiomics model was higher than the AUC of radiologist 3 in the whole set and the training set (all *p* < 0.008), but the difference between two AUCs was not statistically significant in the test set (*p* = 0.03366, adjusted *α* = 0.05/6). For the diagnosis of metastatic LN, the radiomics model showed a slight improvement over the radiologists in the whole set and the training set (whole set: *p*_3_ = 0.004136; training set: *p*_2_ = 0.007323; *p*_3_ < 0.001), but in the test set, there was no statistically significant difference among the radiomics model and radiologists (all *p* > 0.008) (**Supplementary Table S4**). In **Supplementary Table S6**, the radiomics model showed a higher specificity compared with at least two radiologists in all sets in the multiple classification diagnosis of LNTB, cervical lymphoma, reactive LN hyperplasia, and metastatic LN.

The ultrasound radiomics method could provide good capability in differentiating LNTB, cervical lymphoma, reactive LN hyperplasia, and metastatic LN. In the multi-operator, cross-machine, multicenter context, the diagnostic performance of radiomics is comparable and more reproducible compared with those of the skilled radiologists.

## Discussion

The radiomics model developed in our retrospective study showed good accuracy and stability in the multiple classification diagnosis of cervical lymphadenopathy. We collected cervical lymphadenopathy ultrasound images acquired using different instruments in six hospitals with different geographical locations, including three tuberculosis-designated hospitals and three general hospitals. It was both an opportunity and a challenge for the radiomics model training, especially for the multiple classification diagnosis of four causes of cervical lymphadenopathy. Through training and optimization, a radiomics model suitable for the multi-classification diagnosis of cervical lymphadenopathy was trained. The AUC of radiomics was comparable with those of the skilled radiologists who have the best diagnostic performance in cervical lymphadenopathy in the whole, training, and test sets in this study.

Clinical cervical lymph node lesions are common and varied in nature, and the clinical treatments for different types of cervical lymphadenopathy are different, but conventional ultrasound has a certain limit in determining the nature of the lesions. In our study, even though the radiologists had more than 5 years of experience in superficial LN ultrasound examination, the disparity between different radiologists is obvious in the diagnosis of multiple classes of cervical lymphadenopathy. Considering the particularity of tuberculosis in China, doctors in tuberculosis-designated hospitals have more experience in the diagnosis of tuberculosis than in the diagnosis of other lymph node diseases, and they are more sensitive in the differential diagnosis of LNTB. The inter-radiologist consistency for the diagnosis of cervical lymphadenopathy had obvious statistical difference (**Supplementary Table S3**).

However, the diagnostic performance of the radiomics model was consistent between the training set and the test set (**Tables 2**, **3**), which illustrates that the radiomics model could be a stable classifier for cervical lymphadenopathy without constraints of subjective factors and disease distribution among different hospitals. For the diagnosis of LNTB, the AUC of the radiomics model was higher than that of at least one radiologist in all sets (**Supplementary Table S4**). The radiomics model also showed higher sensitivity and specificity compared with radiologists 1–3 in all sets in the diagnosis of LNTB (**Supplementary Tables S5, S6**). For lymphoma, reactive hyperplasia, and metastatic LN, the radiomics model showed higher specificity compared with those of at least two radiologists in all sets (**Supplementary Table S6**). The selected radiomics features from the ultrasound image can discriminate between different lymph node diseases—for example, the first-order, GLRLM, and GLDZM features are selected between LNTB and lymphoma. The first-order features describe the distribution of echo intensities in an image. The GLRLM and GLDZM features quantify the gray-level run-length and gray-level size zone in the image. It might be hard to recognize the high-dimensional features in an ultrasound image by naked eyes. The radiomics model trained for cervical lymphadenopathy in our study could improve the diagnostic ability of ultrasound radiologists and might reduce unnecessary core-needle biopsy in cervical lymphadenopathy.

In recent years, radiomics technology has been widely used in LN diagnosis. Tian et al. developed a radiomics model to predict LN metastasis in patients with confirmed colorectal cancer (27). Another radiomics model was used to manifest atypical primary central nervous system lymphoma (28). Coroller et al. used lymph node radiomic features for predicting the pathological response to neoadjuvant chemoradiation (29). Another study developed a radiomic model to distinguish non-tuberculous mycobacteria from other causes of lymphadenopathy based on CT images from one hospital and obtained an AUC of 89% (30). Considering that this study contains four categories of lymph nodes from six medical centers, it is more complicated and difficult than a binary classification study and much closer to clinical practice, but the classification accuracy is not compromised. Therefore, the method in this study shows great prospects in the multi-classification discrimination of LNTB, cervical lymphoma, reactive LN hyperplasia, and metastatic LN.

In future studies, many improvements should be made to our radiomics model since the current study had some limitations. The sample size of this study was not large enough, and more effective cases need to be accumulated in subsequent studies to optimize the model. The clinical diagnostic validity of this radiomics model will be tested in future clinical trials with more features. Besides this, our study only focused on B-mode ultrasound. However, it is better for a radiomics model to extract more quantitative data from multimodal ultrasound images, including ultrasound elastography, Doppler ultrasound, and contrast-enhanced ultrasound. In subsequent studies, we will collect more data from multimodal images and develop suitable radiomics methods to improve the multi-classification accuracy of cervical lymphadenopathy.

## Data Availability Statement

The original contributions presented in the study are included in the article/**Supplementary Material**. Further inquiries can be directed to the corresponding author.

## Ethics Statement

The studies involving human participants were reviewed and approved by The Second Affiliated Hospital of Zhejiang University School of Medicine. Written informed consent for participation was not required for this study in accordance with the national legislation and institutional requirements.

## Author Contributions

PH and YL designed the study and wrote the manuscript. JC proposed and wrote the method. CZ, QL, and YiqingZ collected the data. YananZ, JL, WX, WL, JZ, and YingZ analyzed the data. QC, YiH, HL, YingH, and GY provided support and comments for the paper. All authors contributed to the article and approved the submitted version.

## Funding

This study was funded by the National Natural Science Foundation of China (numbers 82001818, 82030048, 81527803, and 81901871), National Key R&D Program of China (number 2018YFC0115900), the Zhejiang Science and Technology Project (2019C03077), the Natural Science Foundation of Zhejiang Province (numbers LQ21H180007 and Y16H180019), the Science and Technology Plan of Hangzhou (number 20180533B68), and the Agriculture and Social Development Plan of Hangzhou (number 20190101A09).

## Conflict of Interest

The authors declare that the research was conducted in the absence of any commercial or financial relationships that could be construed as a potential conflict of interest.

## Publisher’s Note

All claims expressed in this article are solely those of the authors and do not necessarily represent those of their affiliated organizations, or those of the publisher, the editors and the reviewers. Any product that may be evaluated in this article, or claim that may be made by its manufacturer, is not guaranteed or endorsed by the publisher.

## Abbreviations

LN, lymph node; TB, tuberculosis; LNTB, lymph node tuberculosis; GLCM, gray-level co-occurrence matrix; GLRLM: gray-level run-length matrix; GLSZM, gray-level size zone matrix; GLDZM, gray-level distance zone matrix; NGTDM, neighborhood gray tone difference matrix; NGDLM, neighboring gray-level dependence matrix; LASSO, least absolute shrinkage and selection operator; SVM, support vector machine; ROC, receiver operating characteristic; ROI, region of interest; ICCs, intraclass correlation coefficients; PPV, positive predictive value; NPV, negative predictive value; CEUS, contrast-enhanced ultrasound.
