# Supplementary material for: Ultrasound-Based Radiomics Can Classify the Etiology of Cervical Lymphadenopathy: A Multi-Center Retrospective Study
Source: Front Oncol. 2022 May 17;12:856605. doi: 10.3389/fonc.2022.856605 (PMC9152112; doi:10.3389/fonc.2022.856605)
Supplement: Supplementary file 1 [file DataSheet_1.docx]

Supplementary Material

**Supplementary Figures**


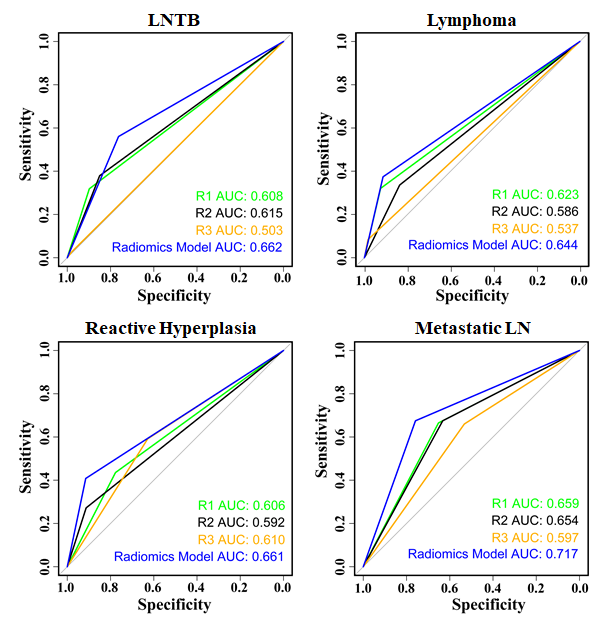


**Supplementary Figure 1. The ROC between radiomics model and senior radiologists (R) in training set**


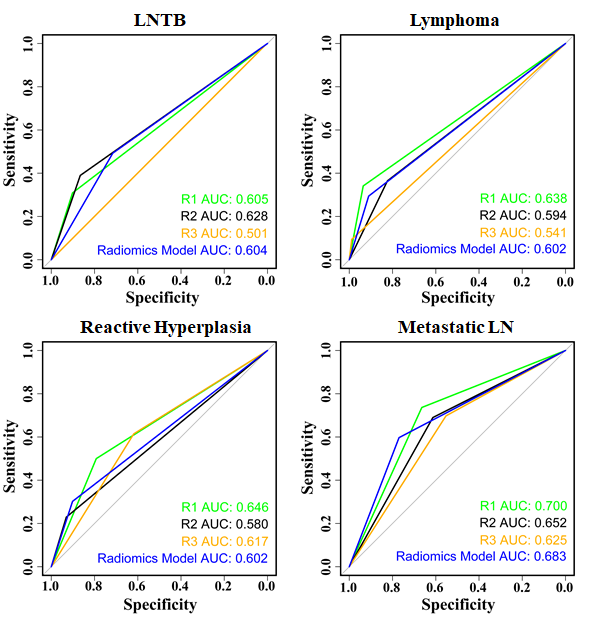


**Supplementary Figure 2. The ROC between radiomics model and senior radiologists (R) in test set**

**Supplementary Table 1. Two algorithm comparation in the diagnosis of cervical lymphadenopathy**

|  | **Training Set (N = 663)** | | | | **Test Set (N = 442)** | | |
| --- | --- | --- | --- | --- | --- | --- | --- |
|  | **Sensitivity** | **Specificity** | | **Accuracy** | **Sensitivity** | **Specificity** | **Accuracy** |
| **Multinomial LASSO-Regression Model** | | | | | | | |
| **LNTB** | **0.543 (0.523–0.564)** | **0.773 (0.752–0.793)** | **0.708 (0.695–0.721)** | | **0.297 (0.274–0.321)** | **0.692 (0.674–0.710)** | **0.581(0.569–0.592)** |
| **Lymphoma** | **0.328 (0.292–0.364)** | **0.921 (0.912–0.929)** | **0.804 (0.797–0.820)** | | **0.131 (0.110–0.152)** | **0.860 (0.843–0.876)** | **0.716 (0.703–0.729)** |
| **Reactive Hyperplasia** | **0.442 (0.418–0.464)** | **0.899 (0.886–0.898)** | **0.793 (0.787–0.799)** | | **0.159 (0.139–0.180)** | **0.839 (0.827–0.852)** | **0.689 (0.680–0.699)** |
| **Metastatic LN** | **0.664 (0.644–0.685)** | **0.751 (0.739–0.763)** | **0.726 (0.716–0.735)** | | **0.397 (0.372–0.421)** | **0.603 (0.589–0.617)** | **0.541 (0.532–0.550)** |
| **LASSO-Vote-SVM Model** | | | | | | | |
| **LNTB** | **0.602 (0.578–0.626)** | **0.765 (0.752–0.779)** | **0.719 (0.709–0.729)** | | **0.480 (0.442–0.518)** | **0.738 (0.713–0.763)** | **0.666 (0.655–0.677)** |
| **Lymphoma** | **0.356 (0.315–0.397)** | **0.930 (0.919–0.940)** | **0.818 (0.811–0.825)** | | **0.255 (0.223–0.287)** | **0.910 (0.895–0.924)** | **0.783 (0.773–0.793)** |
| **Reactive Hyperplasia** | **0.422 (0.390–0.455)** | **0.902 (0.895–0.910)** | **0.798 (0.791–0.805)** | | **0.396(0.360–0.432)** | **0.881 (0.863–0.900)** | **0.773 (0.764–0.784)** |
| **Metastatic LN** | **0.688 (0.669–0.706)** | **0.773 (0.755–0.792)** | **0.748 (0.738–0.758)** | | **0.598 (0.566–0.629)** | **0.723 (0.704–0.742)** | **0.685 (0.676–0.693)** |

| **Supplementary Table 2. The AUC between radiomics model and senior radiologists in whole and training sets.** | | | | | | | | | | |  |
| --- | --- | --- | --- | --- | --- | --- | --- | --- | --- | --- | --- |
| **Data Set** | | **Disease** | | **Radiomics** | | **R1** | | **R2** | | **R3** | |
| **Whole Set** | **LNTB** | | **0.673**  **(0.637–0.710)** | | **0.597**  **(0.559–0.635)** | | **0.611**  **(0.573–0.649)** | | **0.503**  **(0.464–0.540)** | |  |
|  | **Lymphoma** | | **0.623**  **(0.579–0.666)** | | **0.629**  **(0.586–0.672)** | | **0.589**  **(0.546–0.633)** | | **0.537**  **(0.493–0.580)** | |  |
|  | **Reactive Hyperplasia** | | **0.655**  **(0.614–0.695)** | | **0.622**  **(0.581–0.663)** | | **0.588**  **(0.546–0.629)** | | **0.612**  **(0.571–0.654)** | |  |
|  | **Metastatic LN** | | **0.708**  **(0.673–0.743)** | | **0.675**  **(0.639–0.711)** | | **0.653**  **(0.616–0.689)** | | **0.608**  **(0.571–0.646)** | |  |
| **Training Set** | **LNTB** | | **0.662**  **(0.613–0.710)** | | **0.608**  **(0.559–0.658)** | | **0.615**  **(0.565–0.664)** | | **0.503**  **(0.454–0.552)** | |  |
|  | **Lymphoma** | | **0.644**  **(0.588–0.699)** | | **0.623**  **(0.567–0.679)** | | **0.586**  **(0.530–0.642)** | | **0.534**  **(0.478–0.589)** | |  |
|  | **Reactive Hyperplasia** | | **0.661**  **(0.609–0.714)** | | **0.606**  **(0.553–0.660)** | | **0.592**  **(0.538–0.646)** | | **0.610**  **(0.556–0.663)** | |  |
|  | **Metastatic LN** | | **0.717**  **(0.672–0.761)** | | **0.659**  **(0.612–0.705)** | | **0.654**  **(0.607–0.700)** | | **0.596**  **(0.549–0.644)** | |  |

**Supplementary Table 3. The comparison among skilled radiologists in the whole data set**

| **Radiologist (R)** | **LNTB** | **Lymphoma** | **Reactive Hyperplasia** | **Metastatic LN** | **Kappa** | ***p*-value** |
| --- | --- | --- | --- | --- | --- | --- |
| **R1** | **177 (16.0%)** | **134 (12.1%)** | **299 (27.1%)** | **495 (44.8%)** | **0.108** | **< 0.001*** |
| **R2** | **233 (21.1%)** | **225 (20.4%)** | **130 (11.8%)** | **516 (46.7%)** |  |  |
| **R3** | **24 (2.2%)** | **32 (2.9%)** | **469 (42.5%)** | **578 (52.4%)** |  |  |

**Supplementary Table 4. The AUC between radiomics model and senior radiologists**

| **Dataset** | **Disease** | ***p*_1_** | ***p*_2_** | ***p*_3_** | ***p*_4_** | ***p*_5_** | ***p*_6_** |
| --- | --- | --- | --- | --- | --- | --- | --- |
| **Whole Set** | **LNTB** | **0.0003*** | **0.0038*** | **< 0.001*** | **0.3008** | **< 0.001*** | **< 0.001*** |
|  | **Lymphoma** | **0.8204** | **0.0943** | **< 0.001*** | **0.0392** | **< 0.001*** | **0.0048*** |
|  | **Reactive Hyperplasia** | **0.1538** | **< 0.001*** | **0.0693** | **0.0232** | **0.5789** | **0.1727** |
|  | **Metastatic LN** | **0.7774** | **0.0499** | **0.0041*** | **0.1078** | **0.0181** | **0.4171** |
| **Training Set** | **LNTB** | **0.0553** | **0.105** | **< 0.001*** | **0.7143** | **< 0.001*** | **< 0.001*** |
|  | **Lymphoma** | **0.4107** | **0.0293** | **< 0.001*** | **0.1491** | **< 0.001*** | **0.0315** |
|  | **Reactive Hyperplasia** | **0.0762** | **0.0134** | **0.0927** | **0.4634** | **0.8711** | **0.457** |
|  | **Metastatic LN** | **0.0268** | **0.0073*** | **< 0.001*** | **0.8450** | **0.0147** | **0.0301** |
| **Test Set** | **LNTB** | **0.1743** | **0.5057** | **< 0.001*** | **0.2341** | **< 0.001*** | **< 0.001*** |
|  | **Lymphoma** | **0.254** | **0.8123** | **0.0337** | **0.1352** | **< 0.001*** | **0.0673** |
|  | **Reactive Hyperplasia** | **0.2214** | **0.4835** | **0.6991** | **0.009** | **0.2717** | **0.2101** |
|  | **Metastatic LN** | **0.5961** | **0.3027** | **0.0705** | **0.1078** | **0.0181** | **0.4171** |

***p_1_*: radiomics model vs. radiologist 1; *p*_2_: radiomics model vs. radiologist 2; *p*_3_: radiomics model vs. radiologist 3; *p_4_*: radiologist 1 vs. radiologist 2; *p_5_*: radiologist 1 vs. radiologist 3; *p_6_*: radiologist 2 vs. radiologist 3**

***p* < 0.05/6 was defined as statistical significance**

| **Supplementary Table 5: The sensitivity between radiomics model and senior radiologists** | | | | |
| --- | --- | --- | --- | --- |
| **Dataset** | **Disease** | ***p*_1_** | ***p*_2_** | ***p*_3_** |
| **Whole Set** | **LNTB** | **< 0.0001*** | **< 0.0001*** | **< 0.0001*** |
|  | **Lymphoma** | **1** | **0.8149** | **< 0.0001*** |
|  | **Reactive Hyperplasia** | **1** | **< 0.0001*** | **0.0022*** |
|  | **Metastatic LN** | **0.9273** | **0.9219** | **0.7806** |
| **Training Set** | **LNTB** | **< 0.0001*** | **0.001*** | **< 0.0001*** |
|  | **Lymphoma** | **0.3487** | **0.5419** | **< 0.0001*** |
|  | **Reactive Hyperplasia** | **0.7273** | **0.1755** | **0.0023*** |
|  | **Metastatic LN** | **0.9099** | **1** | **0.8124** |
| **Test Set** | **LNTB** | **< 0.0001*** | **0.0009*** | **< 0.0001*** |
|  | **Lymphoma** | **0.6831** | **0.4725** | **0.0009*** |
|  | **Reactive Hyperplasia** | **0.0028*** | **0.4725** | **< 0.0001*** |
|  | **Metastatic LN** | **0.0606** | **0.2913** | **0.2626** |
| ***p_1_*: radiomics model vs. radiologist 1; *p*_2_: radiomics model vs. radiologist 2; *p*_3_: radiomics model vs. radiologist 3**  ***p* < 0.05/6 was defined as statistical significance.** | | | | |

| **Supplementary Table 6. The specificity between radiomics model and senior radiologists** | | | | |
| --- | --- | --- | --- | --- |
| **Dataset** | **Disease** | ***p*_1_** | ***p*_2_** | ***p*_3_** |
| **Whole Set** | **LNTB** | **< 0.0001*** | **0.0006*** | **< 0.0001*** |
|  | **Lymphoma** | **0.1799** | **< 0.0001*** | **< 0.0001*** |
|  | **Reactive Hyperplasia** | **< 0.0001*** | **0.019** | **< 0.0001*** |
|  | **Metastatic LN** | **< 0.0001*** | **< 0.0001*** | **< 0.0001*** |
| **Training Set** | **LNTB** | **< 0.0001*** | **0.0006*** | **< 0.0001*** |
|  | **Lymphoma** | **0.5443** | **< 0.0001*** | **< 0.0001*** |
|  | **Reactive Hyperplasia** | **< 0.0001******* | **1** | **< 0.0001*** |
|  | **Metastatic LN** | **0.0005*** | **< 0.0001*** | **< 0.0001*** |
| **Test Set** | **LNTB** | **< 0.0001*** | **< 0.0001*** | **< 0.0001*** |
|  | **Lymphoma** | **0.2626** | **0.0003*** | **< 0.0001*** |
|  | **Reactive Hyperplasia** | **< 0.0001*** | **0.1845** | **< 0.0001*** |
|  | **Metastatic LN** | **0.0346** | **0.0003*** | **< 0.0001*** |
| ***p_1_*: radiomics model vs. radiologist 1; *p*_2_: radiomics model vs. radiologist 2; *p*_3_: radiomics model vs. radiologist 3**  ***p* < 0.05/6 was defined as statistical significance.** | | | | |

| Supplementary Table 7. The extracted parameters in LASSO model. | |
| --- | --- |
| LNTB vs Lymphoma | LNTB vs Reactive Hyperplasia |
| EnergyCorrelationICM2glrlm_GLVLZESZLGEgldzm_GLNZSNgldzm_SmallDistHighRLgldzm_LargeDistLowGLEmphgldzm_GLnonUnifLowDepLowGLEmphGLnonUnifNormzdDepCountVarm_bccentricitym_pa | CorrelationICM1gldzm_LargeDistEmphgldzm_LargeDistLowGLEmphgldzm_ZoneDistEntropyLowDepLowGLEmphDepCountNonUnifNormzdDepCountVarm_bccentricitym_pa |
| LNTB vs Metastatic LN | Lymphoma vs Reactive Hyperplasia |
| CorrelationICM1ICM2SRELGREHGRELRLGEglrlm_GLVSZELZEGLNNZSNZSNNZPGLVEntropygldzm_SmallDistEmphgldzm_LowGLCountRmphgldzm_SmallDistHighRLgldzm_LargeDistLowGLEmphgldzm_ZonePercentagegldzm_ZoneDistVargldzm_ZoneDistEntropyContrastBusynessHighDepLowGLEmphDepCountNonUnifNormzdDepCountVarDepCountEnergym_varm_medm_bccentricitym_orientationm_pa | CorrelationClusterprominenceICM1ICM2SRELRELGRESRHGEglrlm_GLVSZEGLVgldzm_SmallDistEmphgldzm_LowGLCountRmphgldzm_SmallDistHighRLgldzm_LargeDistHighGLEmphgldzm_ZoneDistNonUnifgldzm_GLVargldzm_ZoneDistEntropyBusynessLowDepLowGLEmphLowDepHighRLHighDepLowGLEmphDepCountEnergym_varm_bccentricity |
| Lymphoma vs Metastatic LN | Reactive Hyperplasia vs Metastatic LN |
| LGRESRLGESZHGEgldzm_LargeDistHighGLEmphDepCountNonUnifm_varm_bccentricitym_pa | CorrelationClusterprominenceICM2InvDifMomentIDMNIDNSRELREGLNSRLGEglrlm_GLVSZLGEGLVgldzm_SmallDistEmphgldzm_SmallDistHighRLgldzm_LargeDistLowGLEmphgldzm_GLnonUnifNormzdgldzm_ZoneDistNonUnifgldzm_GLVargldzm_ZoneDistVarBusynessLowGLCountRmphm_varm_bccentricitym_orientationm_pa |
